# Augmented Reality System for Digital Rectal Examination Training and Assessment: System Validation

**DOI:** 10.2196/18637

**Published:** 2020-08-13

**Authors:** Theerapat Muangpoon, Reza Haghighi Osgouei, David Escobar-Castillejos, Christos Kontovounisios, Fernando Bello

**Affiliations:** 1 Faculty of Medicine, Department of Surgery and Cancer Imperial College London London United Kingdom

**Keywords:** Augmented Reality, Digital Rectal Examination (DRE), Magnetic Tracker, Pressure Sensor, Medical Education, Usability

## Abstract

**Background:**

Digital rectal examination is a difficult examination to learn and teach because of limited opportunities for practice; however, the main challenge is that students and tutors cannot see the finger when it is palpating the anal canal and prostate gland inside the patients.

**Objective:**

This paper presents an augmented reality system to be used with benchtop models commonly available in medical schools with the aim of addressing the problem of lack of visualization. The system enables visualization of the examining finger, as well as of the internal organs when performing digital rectal examinations. Magnetic tracking sensors are used to track the movement of the finger, and a pressure sensor is used to monitor the applied pressure. By overlaying a virtual finger on the real finger and a virtual model on the benchtop model, students can see through the examination and finger maneuvers.

**Methods:**

The system was implemented in the Unity game engine (Unity Technologies) and uses a first-generation HoloLens (Microsoft Inc) as an augmented reality device. To evaluate the system, 19 participants (9 clinicians who routinely performed digital rectal examinations and 10 medical students) were asked to use the system and answer 12 questions regarding the usefulness of the system.

**Results:**

The system showed the movement of an examining finger in real time with a frame rate of 60 fps on the HoloLens and accurately aligned the virtual and real models with a mean error of 3.9 mm. Users found the movement of the finger was realistic (mean 3.9, SD 1.2); moreover, they found the visualization of the finger and internal organs were useful for teaching, learning, and assessment of digital rectal examinations (finger: mean 4.1, SD 1.1; organs: mean 4.6, SD 0.8), mainly targeting a novice group.

**Conclusions:**

The proposed augmented reality system was designed to improve teaching and learning of digital rectal examination skills by providing visualization of the finger and internal organs. The initial user study proved its applicability and usefulness.

## Introduction

Digital rectal examination (DRE) is a physical examination for detecting rectal and prostate abnormalities. Focusing on prostate examination, DRE requires that an index finger to palpate the prostate gland to detect abnormalities in gland size, tenderness, and surface texture. Even though it is a recommendation of the American Cancer Society to perform DRE to screen and detect prostate cancer in patients with colorectal symptoms, multiple studies [1,2] have found that, during their final year, medical students are not confident in their abilities to perform the examination. The lack of confidence in performing DRE among medical students has mainly been attributed to not having adequate practice in medical schools [1].

**Figure 1:**
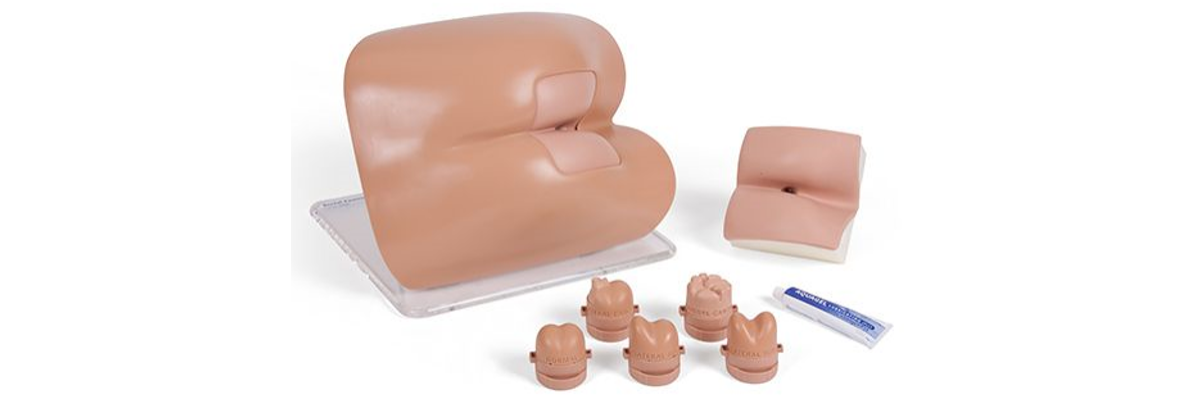
A standard benchtop model used in medical schools for teaching and practicing digital rectal examination.

A standard method to train DRE skills is to practice on a benchtop model. This model is a plastic human mannequin with skin-like rubber to represent the rectal canal with several plastic replaceable prostate models (Figure 1). Even though students can touch and feel the prostate gland through the rubber rectum, no visualization of finger movement or internal organs can be obtained because it lacks transparency. Similarly, this model does not provide enough information to examiners to assess the techniques used by students to perform DRE.

There have been several attempts to improve the visualization of DRE on a benchtop model. Early attempts include a training system using virtual reality technology, together with a Phantom haptic device [3] which displayed a simplified 3D model of kidneys, rectum, bladder, and prostate along with the virtual representation of the examining finger on the 2D monitor. This system was evaluated against the rubber model, and it was concluded that it could be a new way to train DRE if the realism of the haptic system was improved [3]. In another study [4], a similar approach used a haptic interface for palpating the prostate gland. In follow-up work and to improve the design of haptic-based learning tools, Granados et al [5] conducted a study to better understand palpation techniques of experts while conducting DRE on a real subject. Dobson et al [6] proposed a system using virtual reality technology to visualize the anatomy in the pelvic area [6]. For their system, the user had to wear special glasses to view the model in 3D which was displayed on the 67-inch×50-inch screen (VR ImmersaDesk). It was shown that the system helped medical students in gaining more understanding of the anatomy and results in better exam scores [6]. Rissanen et al [7] introduced Annotated Simulation Records for DRE, which focused on using virtual reality technology to reveal useful data from the sensor during DRE practice. In this system, the urologist selected the most useful parameters to be annotated to help in teaching DRE. This system was evaluated by medical students, and it was found that the numerical annotations helped them learn faster than verbal feedback. Balkissoon et al [8] introduced a DRE training system that consisted of a physical benchtop model and a 2D screen for visualizing the DRE in which multiple sensors were attached to the prostate gland in the benchtop model to measure user applied pressure on various locations. Visual information, including applied force, palpated area, and palpation at each location, was displayed during the examination. The study [8] showed that the sensors could help the instructor to observe and assess the performance of medical students performing DREs. In another model [9], a similar concept, embedding pressure sensors in the model, was followed.

Displaying information, such as finger position and pressure on a 2D screen, was demonstrated to be beneficial in understanding and performing DRE; however, the user experience was not ideal due to the lack of colocation between the benchtop model and the display.

In this paper, we present an augmented reality system for DRE visualization. It uses sensors attached to the examining finger to track its maneuvers and to monitor applied pressure. It also displays the DRE and essential information overlaid on the real benchtop model using an augmented reality device. The main goal was to improve the user experience of a widely available benchtop model. The paper first describes the visualization of the examining finger and internal organs, the step-by-step guidance for DRE, and the performance recording feature. Results, including performance measures and feedback from clinicians and medical students, are then reported, followed by discussion and conclusions.

## Methods

### Hardware and Software

The proposed augmented reality system is used as an extension to a standard Rectal Examination Trainer (Mk2, Limbs & Things Inc) benchtop model (Figure 1, [10]). The model is a semirealistic representation of the buttocks, anus, and rectum, allowing for the practice of diagnostic skills associated with rectal examination. It includes additional rectal examination perineum, which contains two rectal pathologies (polyp and carcinoma). For prostate examination, five interchangeable prostates are provided: normal, bilateral benign, unilateral benign, bilateral carcinoma, and unilateral carcinoma. The model can be used for both digital examination of prostate and rectum, as well as for the insertion and use of anoscope and proctoscope.

**Figure 2:**
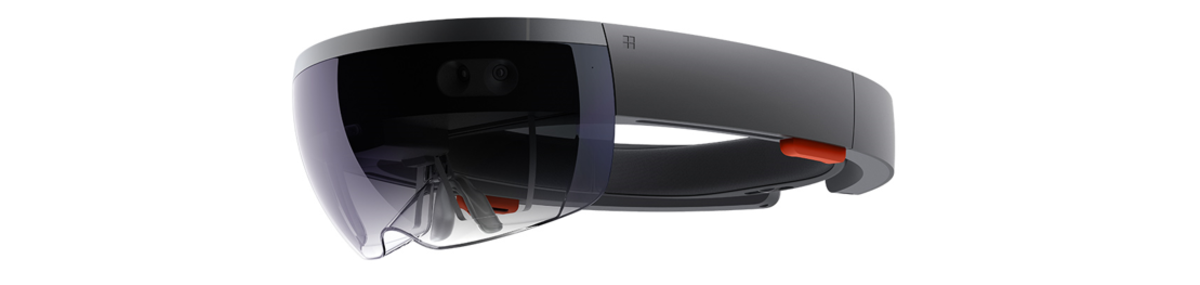
Microsoft HoloLens, a head-mounted augmented reality device to display 3D virtual objects.

We used the HoloLens (version 1, Microsoft Inc) as an augmented reality head-mounted display (Figure 2). The HoloLens is immersive and see-through to help the user perceive the environment as realistic. It also enables interactions with holographic content. The HoloLens features an inertial measurement unit (accelerometer, gyroscope, and magnetometer), 4 environment understanding sensors (2 on each side), an energy-efficient depth camera with a 120°×120° angle of view, a 2.4-megapixel photographic video camera, a 4-microphone array, and an ambient light sensor [11].

HoloLens has been used in various medical simulations; VimedixAR (CAE Inc) was the first ultrasound simulator to integrate a HoloLens [12]; with this system, health care professionals manipulate representations of realistic anatomical parts and view the ultrasound beam in real time as it passes through human anatomy. With CAE LucinaAR, clinical learners can view 3D holograms of a fetus, as it descends the birth canal, and learn to manage complex deliveries [13]. HoloPatient (Microsoft Inc) is a mixed-reality learning tool for nursing, allied health, and medical schools that delivers simulated patient experiences [14]. Learning Heart (Spheregen) is a HoloLens application that assists students in understanding the physiology of the heart [15].

The 3D software in our system was developed using the Unity games engine (version 2018.3.7f1; Unity Technologies) [16] cross-platform authoring tool for creating 3D content.

### Real-Time Performance Visualization

**Figure 3:**
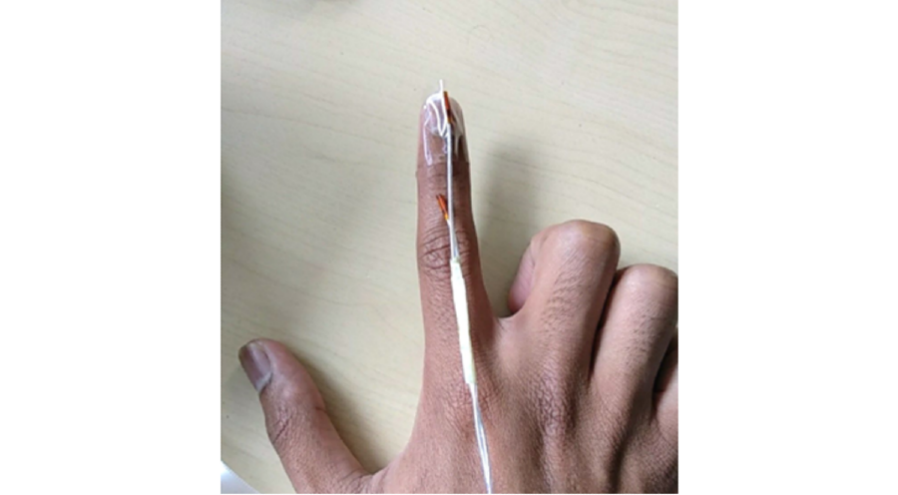
A Trakstar magnetic positioning sensor attached to the examining finger.

**Figure 4:**
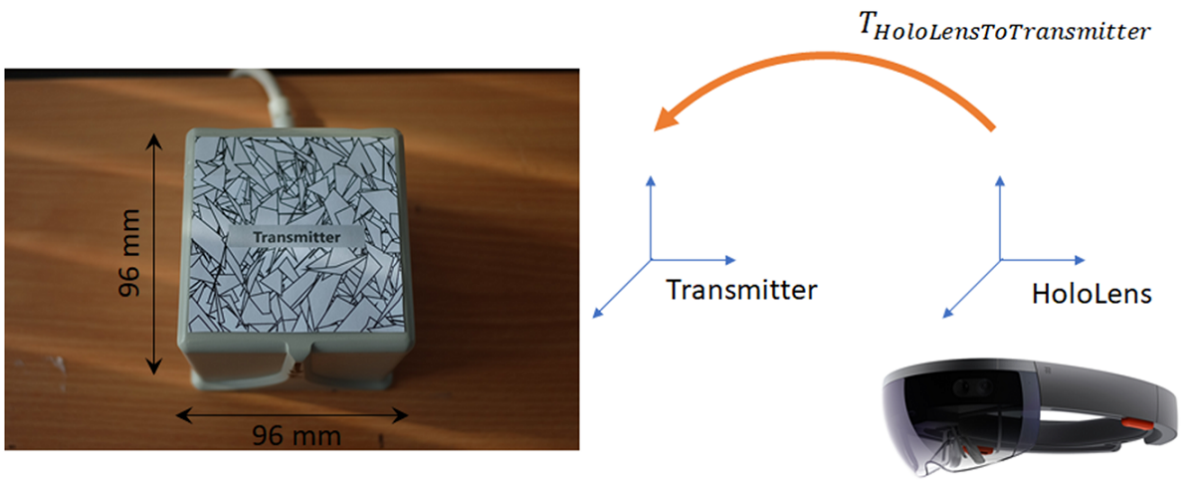
Conversion process of the position tracking coordinates to real-world (HoloLens) coordinates using Vuforia image tracking.

**Figure 5:**
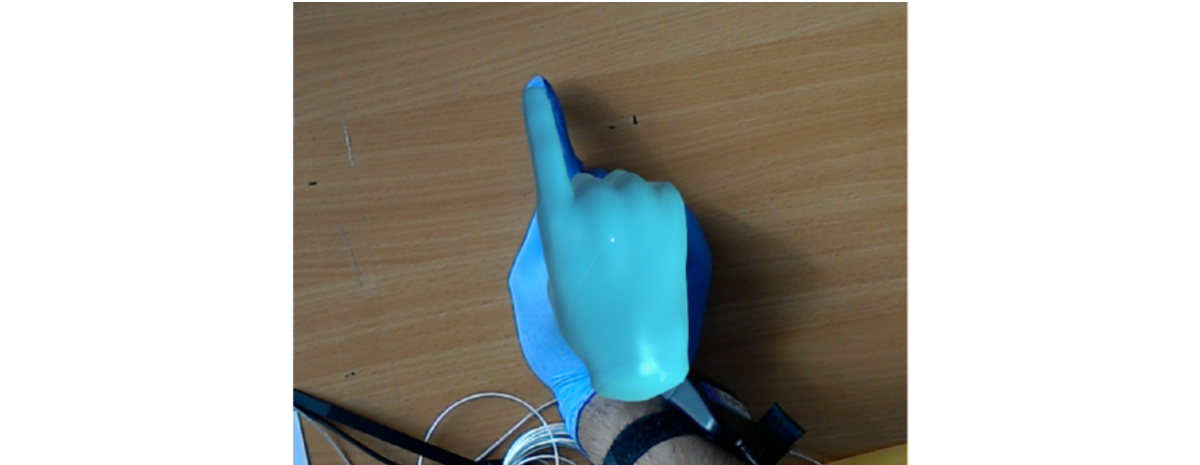
The virtual finger in blue overlaid onto the real examining finger with a blue medical glove.

To track and show the movement of the examining finger inside the benchtop model during the examination, we used a Trakstar magnetic tracking system (Northern Digital Inc) to obtain the position and orientation (pose) of the examining finger in real time, due to its ability to operate without line-of-sight [17]. It consists of a midrange magnetic field transmitter and a 6 degrees-of-freedom receiver (model 180), and it was connected to an electronics unit for amplification and digitization. The 6 degrees-of-freedom sensor has a position accuracy of 1.40 mm RMS and orientation accuracy of 0.50° RMS. The combination of the transmitter and the receiver allows tracking within a 30×40×30 cm^3^ zone, which is large enough for tracking the examining finger inside the benchtop model. The sensor was attached to the finger using thin tape (Figure 3, [17]). Sensor data were read at 40 Hz by the computer via an API using a previously developed C++ plug-in [18]. Once read, sensor data was transferred to the HoloLens via Wi-Fi. Position and orientation were transformed into the real-world coordinate system with the help of Vuforia Engine (version 8.1.7) [19]. The transformation was achieved by using the HoloLens to track the pose of the image target in world coordinates (Figure 4). This transformation enabled synchronization and overlay of the virtual finger on the tracked real finger, and for the result to be seen through the HoloLens (Figure 5).

**Figure 6:**
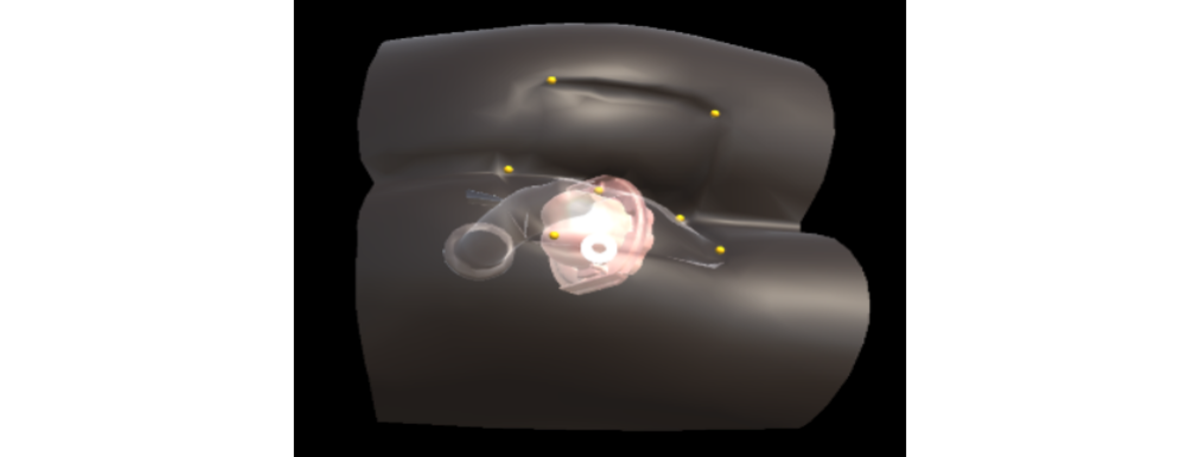
3D virtual benchtop model.

**Figure 7:**
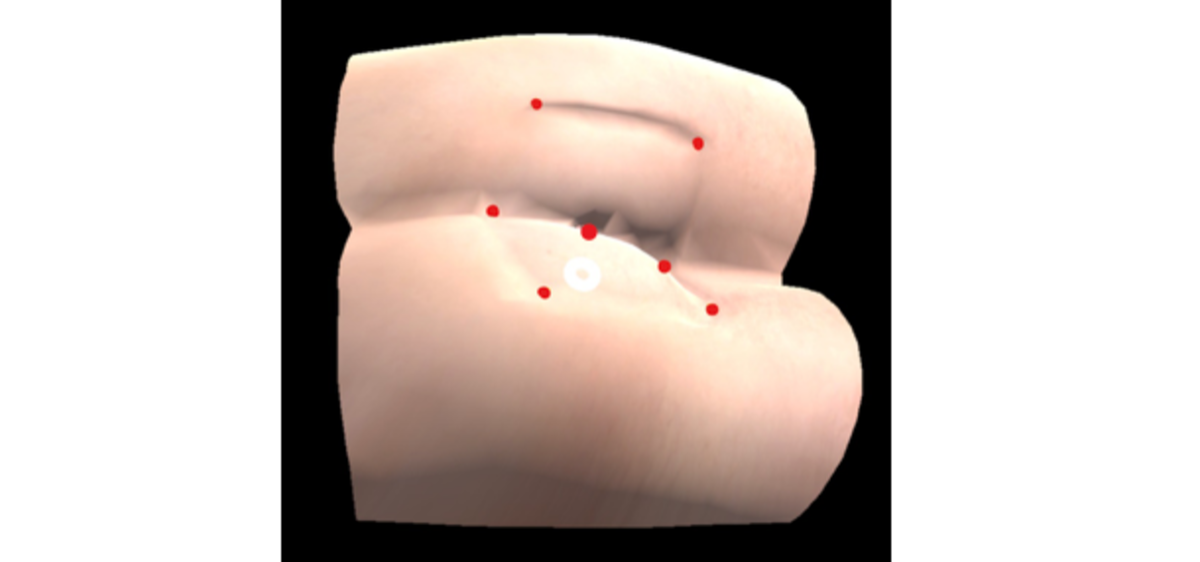
Red landmarks on a benchtop model used as inputs to the iterative closest point algorithm.

**Figure 8:**
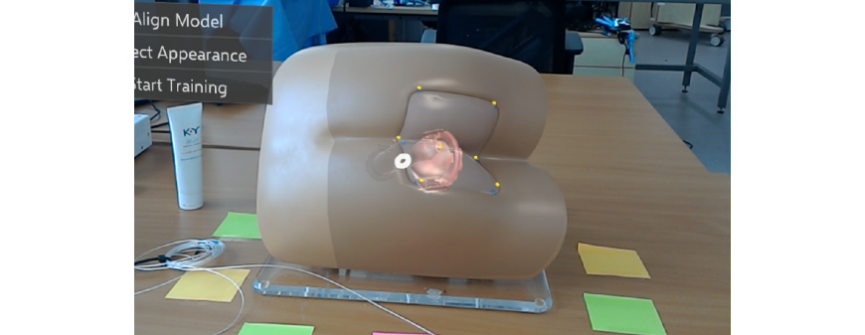
A virtual transparent benchtop model with a 3D virtual prostate inside is aligned with the physical benchtop model.

**Figure 9:**
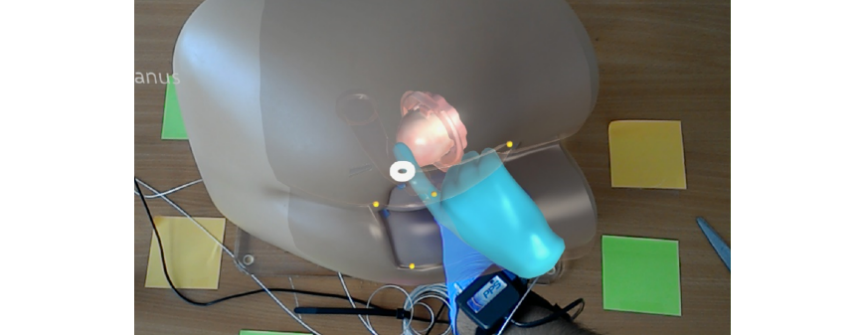
Real-time visualization of digital rectal examination using the proposed augmented reality system.

To visualize the internal components of the benchtop model and to overlay the relevant virtual anatomy, it was necessary to first align the virtual model (Figure 6) with the physical benchtop model. For this purpose, the iterative closest point algorithm was used [20]; it takes the position of 7 anatomical landmarks as an input (Figure 7) and yields a transformation matrix. This matrix is used to rotate and translate the virtual benchtop model and virtual internal organs to align with the physical benchtop model (Figure 8). Once aligned, the user can visualize the movement of the examining finger and the model by directly looking at the model with the HoloLens (Figure 9).

**Figure 10:**
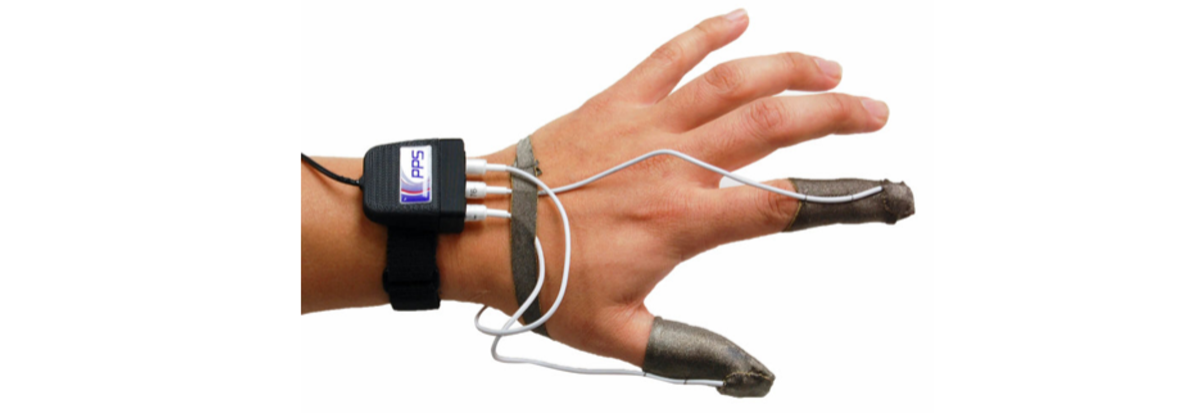
A FingerTPS pressure sensor. It measures pressure applied during the prostate palpation.

**Figure 11:**
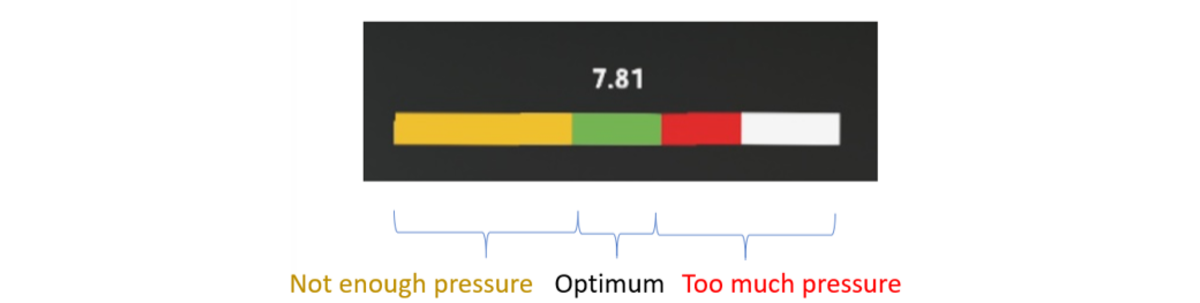
Real-time visualization of force applied by the examining finger.

A FingerTPS force sensor [21] was also attached to the examining finger to estimate the pressure applied to the prostate during palpation. The force sensor is flat and thin and can be worn under a surgical glove (Figure 10, [21]). The force sensor data were transferred to the HoloLens using the same process as for the pose information from the tracking sensor. A force visualizer, represented as a color bar with 3 regions showing different levels of pressure applied to the prostate gland, was displayed to provide real-time feedback (Figure 11).

### Internal Organ Labeling

Labels were used for displaying relevant information regarding internal anatomy inside the benchtop model; however, since there were several anatomical structures inside a small area, this made it difficult to label the anatomy in an effective way (in a way that avoids overlap with other labels or with the anatomy). Also, since our system allowed users to look at the anatomy from different perspectives, labels needed to be dynamically positioned. To address these issues, we implemented a view management system, capable of resolving occlusion among labels. Our labeling system used the labeling technique suggested by Tatzgern [22] (overcoming occlusion by limiting update rates, ie, not continuously moving the labels to separate them, but only when they occlude each other) combined with the theory by Hartzmann [23] to ensure that the labels were always near the object and that the lines from labels to objects did not cross each other. To achieve these criteria, the following steps are iteratively applied until all occlusions are resolved: (1) Place the label near the referenced object on the surface of the sphere, centered at the center of mass of the benchtop model. (2) Iterate through all labels to find all those that may be occluded by another label, establishing the side of the occlusion. (3) Move the occluded label to the opposite side to resolve the occlusion.

**Figure 12:**
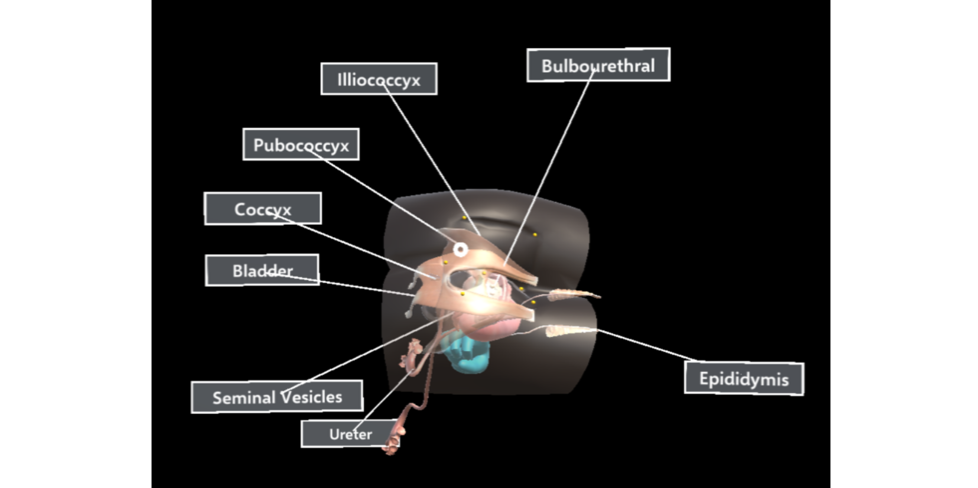
Occlusion between labels is resolved by pushing them apart along the circumference of the sphere, whose center is at the center of mass of the benchtop model.

The labels were created using a ToolTip component of Unity, provided by the Microsoft Mixed Reality Toolkit (version 2.0.0 RC2) [24]. This component can rotate the label to always face the user, making it more readable (Figure 12).

### Step-By-Step Guidance

**Figure 13:**
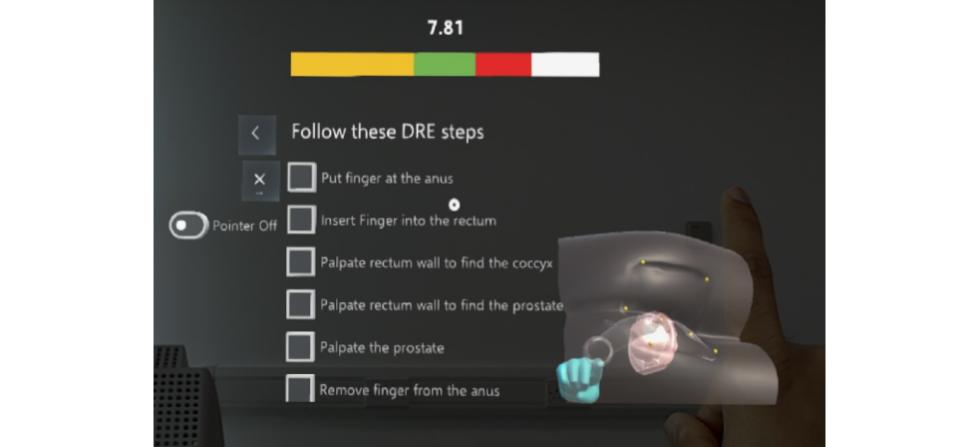
Step-by-step guidance next to the virtual benchtop model. DRE: digital rectal examination.

Our augmented reality system incorporated step-by-step guidance to help trainees follow the correct steps and trajectory during the examination. Steps were extracted from the cognitive task analysis study performed by Low-Beer et al [25]. The system automatically tracked the position of the finger inside the rectal canal and checked whether the trainee had correctly followed the step. The user interface was designed to be readable and to require the least possible head movement to see all the contents (Figure 13), despite the narrow field of view of the HoloLens display.

### Performance Recording

In addition to step-by-step guidance, our augmented reality system also allowed recording and playback of the examination. The pose of the examining finger and the benchtop model were recorded so that, when played back, the virtual examining finger and the virtual benchtop model could be accurately displayed on the HoloLens. This feature is useful trainees as they can observe experts repeatedly, or they can analyze their own performance. It can also be used by an examiner to assess student performance from different angles.

### Model Alignment Evaluation

The accuracy of the model alignment system was evaluated by performing an alignment task five times by an experienced user. After each alignment task was performed, the positions of each landmark on both the physical and virtual benchtop models were then measured using the magnetic tracking sensor. The error of the alignment system was then calculated as a distance between a point on the virtual model and a corresponding point on the physical model.

### Pilot User Study

An initial validation study was conducted. Clinicians (n=9; 6 men, 3 women; mean 37.8, SD 5.4 years of age) who routinely perform DRE, and medical students (n=10; 7 men, 3 women; mean 21.8, SD 2.4 years of age) were recruited. The study was approved by the National Health Service Patient Safety Agency Research Ethics Committee (09/H0701/68). Before the study, a consent form was signed by each participant. During the study, participants were asked to perform DREs on the benchtop model with the augmented reality system, wearing both the position tracking sensor and the pressure sensor under a standard surgical glove. They were also asked to pay attention to the information displayed on the HoloLens, such as the force bar and the guidance panel. Once finished, they were asked to watch the recorded performance. Afterward, participants were asked to complete an online questionnaire using a 5-point Likert scale from 1 (definitely disagree) to 5 (definitely agree) regarding the usefulness of the system (Table 1).

**Table 1:** Questions assessing the usefulness of the system.

Number	Question
Q1	The record of the expert's performance would be useful for DRE^a^ teaching or learning
Q2	The movement of the examining finger would be useful for DRE training
Q3	Being able to visualize the internal organs in the benchtop model could help a trainee better understand DRE
Q4	The real-time visualization of force applied to the model would be useful for DRE training
Q5	The step-by-step guidance would be useful for DRE training
Q6	The movement of the examining finger inside the model is realistic and accurate
Q7	The virtual representation of benchtop model can be aligned accurately on the physical model
Q8	The AR^b^ system is easy to use and understand
Q9	The AR system requires minimum movement to operate
Q10	During the performance, you feel tired or fatigued
Q11	The AR system can enhance current teaching and learning of DRE
Q12	I would recommend the AR system to be integrated into the medical curriculum

^a^DRE: digital rectal examination.

^b^AR: augmented reality.

## Results

The augmented reality prototype system ran stably and achieved a frame rate of 60 fps, which is the highest possible frame rate of the HoloLens and Unity. The alignment between the virtual and real fingers and between the virtual and benchtop models was acceptable when visually inspected. The average alignment error of each landmark was an overall average of 1.73, 2.91, and 1.91 mm in the x, y, and z directions, respectively, or a root mean square of 3.9 mm (Table 2).

**Figure 14:**
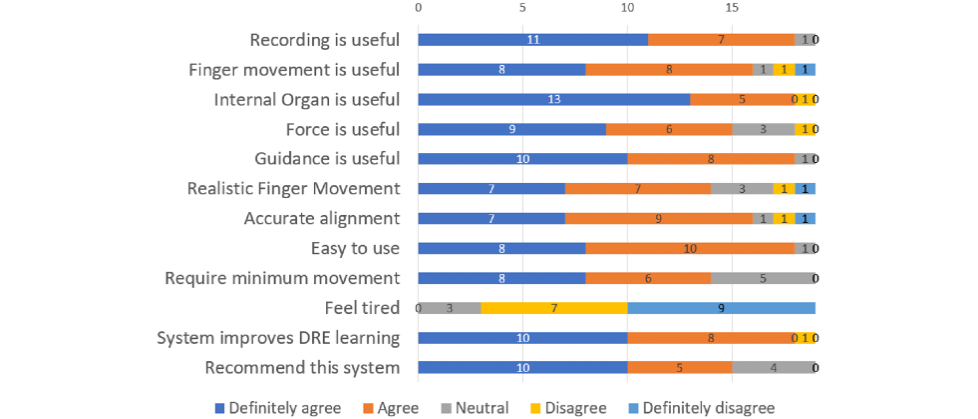
Results from the Likert-scale questionnaire. The numbers and hence the length of each bar indicate the number of participants choosing that rate. DRE: digital rectal examination.

The usefulness of the system was assessed from the answers given to the questionnaire (Figure 14). Most participants would recommend this system to be integrated into medical school curriculum (mean 4.3, SD 0.8).

**Table 2:** Model alignment error.

Landmark	Alignment error (mm)		
	x, mean (SD)	y, mean (SD)	z, mean (SD)
1	0.46 (0.72)	1.96 (2.49)	0.48 (0.34)
2	2.38 (0.29)	2.24 (0.39)	2.02 (0.25)
3	2.04 (0.25)	0.78 (0.29)	4.38 (0.86)
4	1.02 (0.45)	5.68 (1.85)	3.06 (0.62)
5	1.18 (0.62)	6.40 (0.32)	0.96 (0.35)
6	1.90 (0.61)	1.20 (0.40)	1.92 (0.50)
7	2.98 (0.67)	2.12 (0.27)	0.52 (0.66)
Mean	1.73 (0.95)	2.91 (2.37)	1.91 (1.72)

According to the results, some features were more useful than others. For example, most participants agreed that performance recording was useful (mean 4.5, SD 0.6). Regarding the real-time feedback feature, most participants agreed that the visualization of an examining finger was useful (mean 4.1, SD 1.1). The highest score was achieved for the usefulness of the visualization of internal organs (mean 4.6, SD 0.8). Step-by-step guidance was also one of the most highly rated features (mean 4.5, SD 0.6). Regarding the usability of the system, participants responded that it was easy to use (mean 4.4, SD 0.6), and they did not feel fatigued after using the system (mean 1.7, SD 0.7). The alignment of the virtual benchtop model with the real benchtop model was also rated as very accurate (mean 4.1, SD 1.1).

**Figure 15:**
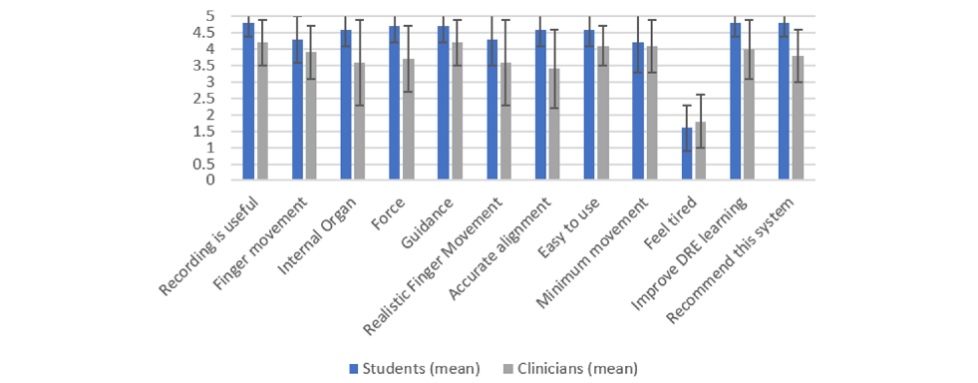
Comparison between questionnaire results from medical students and clinicians (1 = definitely disagree, 5 = definitely agree) regarding the usefulness of the augmented reality system. DRE: digital rectal examination.

The results from both groups, medical students and clinicians, are given in Figure 15. The scores obtained from the students were higher than those from the clinicians for all features (mean 0.63, SD 0.41).

## Discussion

### Principal Findings

The results showed that medical students and clinicians were interested in the system and recognized the value of visualization of DRE, performance recording, and step-by-step guidance in improving the learning and teaching of DRE skills. Medical students could visualize the movement of the finger and the pressure applied to the prostate. This visualization and sensor data, combined with the step-by-step guidance, allowed them to receive feedback in real time while performing DRE. Using the record-and-playback feature, students could not only rewatch examinations performed by an expert, but examiners could also review student performance from multiple viewing angles.

Most users reported that the HoloLens and the tracking sensors were comfortable to wear. Even with a narrow field of view, the user interface did not appear cluttered, facilitating navigation through the menu and visualization of the performance, while at the same time facilitating step-by-step guidance on the left-hand side of the display. The model alignment was also perceived as accurate which demonstrated the use of the iterative closest point algorithm, and the readings from the electromagnetic positioning sensor were valid.

User experience was improved by displaying all information on a head-mounted augmented reality display that also allowed the overlaying of the virtual finger on the real finger. This colocation allowed trainees to avoid having to change visual focus from the benchtop model to a separate display.

When comparing the feedback from medical students to the feedback from clinicians, it was observed that the mean ratings from medical students were higher than those from clinicians for all questions, with an average difference of 20%. A likely explanation is that experienced clinicians had higher expectations, and they were comparing the quality of the simulation with that of real cases because of their experience performing DREs. Having less experience, students would be more likely to require visualization and guidance, while clinicians would not because tactile feedback was adequate, given their level of experience. In addition, it may also reflect the increased acceptability and interest in this type of technology by younger generations.

While the proposed system was demonstrated to be beneficial, it has some limitations. Wearing and adjusting the HoloLens properly can take some time and require assistance. In addition, operating and interacting with the HoloLens by using gestures such as pinching, also requires practice. Regarding the sensors, properly wearing the tracking and pressure sensors is crucial, and at the moment, requires the presence of an assistant to be properly placed on the examining finger. In terms of implementation, the current alignment between the virtual and the physical benchtop model was done manually through 7 landmarks; however, automatic alignment would be faster, more accurate, and more convenient. With respect to features, a scoring system with real-time feedback would be valuable for teaching and assessing DRE skills. Apart from these, the benchtop model itself was reported to be much stiffer than real patients and generally unrealistic with limited anatomical landmarks.

### Conclusions

This paper presents an augmented reality system for teaching and learning DRE that can be used with widely available benchtop models. It was designed to assist both trainees and teachers in learning, teaching, and practicing DRE, as well as to allow examiners to assess student technique. With colocation of the virtual and real models, students only need to focus on the benchtop model to visualize all important information. Even though the results from the user study are positive, further research is needed to evaluate the system. This would include more robust quantitative analysis with a larger number of participants and varying levels of experience. The augmented reality system could be improved by using the second version of the HoloLens which offers a larger field of view and resolution, and it could also be improved by using a haptic-based instead of standard benchtop model so that a wider variety of prostate glands and abnormalities may be generated and used during the examination. Such a model would also be able to directly track finger movement and estimate pressure applied without the need for external sensors. Finally, formative and summative assessment of DRE performance will be an important component of the next version of our system.

## Abbreviations

DRE: digital rectal examination
RMS: root mean square
